# Knowledge and perception of nosocomial infections among patients in a Nigerian hospital

**DOI:** 10.1038/s41598-023-47661-0

**Published:** 2023-11-18

**Authors:** Olawale Oni, Edidiong Orok, Zainab Lawal, Tolulope Ojo, Tunrayo Oluwadare, Toba Bamitale, Boluwaji Jaiyesimi, Alice Akinjisola, Titilayo Apara

**Affiliations:** 1 Department of Public Health, College of Medicine and Health Sciences, Afe Babalola University, Ado-Ekiti, Ekiti State Nigeria; 2 Department of Clinical Pharmacy and Public Health, College of Pharmacy, Afe Babalola University, Ado-Ekiti, Ekiti State Nigeria; 3 Department of Mathematical and Physical Sciences, College of Science, Afe Babalola University, Ado-Ekiti, Ekiti State Nigeria

**Keywords:** Infectious diseases, Bacterial infection

## Abstract

Nosocomial infections are infections that are a leading cause of morbidity and mortality among hospitalized patients, and can lead to higher healthcare costs and longer hospital stays in both developed and developing countries. The objectives of the study were to identify the level of knowledge and perception of patients on nosocomial infection, and to determine the factors affecting the knowledge and perception of patients on nosocomial infection in Federal Medical Centre, Ebute-Metta, Lagos (FMC). A cross-sectional research design was used in carrying out the research among in-patients of FMC where an interview-based semi-structured questionnaire was used for data collection. Patients’ knowledge was categorised as good (≥ 70% score), fair (50–69%) and poor (< 50% score) while perception was grouped as positive and negative. Independent sample T-test and One-way Analysis of Variance was used to assess statistical difference in knowledge scores between categorical variables with 2 and 3 groups respectively. A total of 102 patients gave consent to participate in this study of which 46.1% were male, 27.5% were aged between 38 and 47 years and 69.6% were married. About 24% did not know what is meant by nosocomial infections while 53.9% stated that all hospital-acquired infections are preventable. Less than 19% strongly disagreed that making alcohol rubs mandatory for all visitors would decrease the incidence of nosocomial infections while all strongly agreed that if left untreated, such infections can become life-threatening. Overall, 15.7% showed good knowledge while most patients showed fair knowledge (71.6%) and a negative perception (51%) of nosocomial infections. There was a significant difference in patients’ knowledge of nosocomial infections based on age (0.012). There was also a statistically significant association between age and perception of patients towards nosocomial infections (p = 0.031). This study showed that most patients had fair knowledge as well as negative perception towards nosocomial infections. Age, had an influence on patients’ perception and knowledge of nosocomial infection.

## Introduction

Nosocomial infections, often known as hospital acquired infections, are those infections that a patient contracts while being treated in a hospital^1^. These infections can occur during healthcare delivery for other diseases as well as after patients have been discharged. They also include occupational infections among medical personnel^2^. Invasive devices used in modern health care, such as catheters and ventilators, are linked to these infections^3^. With an increase in infections, there is an increase in hospitalization, long-term disability, antimicrobial resistance, socioeconomic disruption, and mortality rate. Due to inadequate surveillance systems and lack of control methods, there is little information on the burden of nosocomial infections. This is significant because when patients are undergoing treatment for other medical conditions, there is a high likelihood of them contracting respiratory infections, which can complicate the accurate assessment of the prevalence of any nosocomial infection within a primary care facility^4^.

Despite improvements in the knowledge and ability to control these illnesses, they nevertheless pose a serious threat to global public health. Even the best clinical care can be useless if patients contract additional infections while they are being treated in the hospital. Nosocomial infections occur during stays of 48 h or longer, leading to the use of the 48-h criterion in several epidemiological surveillance systems ^5^.

Microorganisms such as *Streptococcus* spp., *Acinetobacter* spp., *enterococci, Pseudomonas aeruginosa*, Coagulase negative *staphylococci*, *Staphylococcus aureus*, *Bacillus cereus*, *Legionella*, and members of the *Enterobacteria* family are among the organisms that are frequently involved in nosocomial infections. These microorganisms can be spread from person to person, via shared objects and surfaces, the environment, contaminated water and food, diseased people, and contaminated healthcare workers' skin^6^. The infection may have come from the outside environment, another sick patient, or potentially infected employees. Sometimes the microorganism comes from the patient's own skin microbiota and becomes opportunistic as a result of surgery or other treatments that undermine the skin's barrier of defence. Even though the patient may have acquired the illness through their own skin, it is still regarded as nosocomial because it arises in a medical facility^7^.

Nosocomial infection affects a large number of patients worldwide, significantly increasing mortality rates and financial losses. The World Health Organization (WHO) estimates that each year, hundreds of millions of patients experience nosocomial infections which are a major source of illness and mortality^8^. However there are few reliable data sources available on the occurrence of nosocomial infections globally. According to WHO estimates, approximately 15% of all hospitalized patients have these infections^8^. Nosocomial infections account for 4–56% of all neonatal deaths, with a 75% incidence rate in South-East Asia and Sub-Saharan Africa^2^. In high-income countries, the incidence ranges between 3.5 and 12%, whereas it ranges between 5.7% and 19.1% in middle- and low-income countries^2^. Also, in the developed world, the prevalence is reported to be 15% among hospitalized patients, but as high as 37% among patients admitted to the Intensive Care Unit^9^.

The prevalence of nosocomial infections in Africa is relatively high, but the available data are limited. The prevalence of nosocomial infections in Africa was estimated to be 12.2% [95% confidence interval (CI), 9.8–14.8%] in a systematic review and meta-analysis of studies carried out between 2000 and 2018^10^.

Similar findings have been reported in Nigeria, where these infections are a leading cause of morbidity and mortality among hospitalized patients, and they can lead to higher healthcare costs and longer hospital stays^11^. A meta-analysis of studies conducted in Nigeria revealed that the overall prevalence of nosocomial infections in the country was 20.2%^11^. Surgical site infections, urinary tract infections, and bloodstream infections were the most common types of infections reported.

Nosocomial infections are prevalent in Nigeria due to a variety of factors, including poor infection prevention and control practices, insufficient resources, and a lack of awareness, poor perception and poor knowledge among healthcare workers and patients^11^. Furthermore, the overuse of antibiotics in Nigeria has resulted in the emergence of antibiotic-resistant bacteria, which can complicate the management of nosocomial infections even further^12^. According to Efstathiou et al., (2019), one of the primary reasons for the high incidence of nosocomial infections in hospital settings is a lack of knowledge and awareness among patients^13^. Patients who are well-informed and knowledgeable about the risks and prevention of nosocomial infections are more likely to take precautions.

The reduction of nosocomial infections is a global priority, and a number of approaches have been suggested to do so, including the use of infection prevention and control measures, prudent antibiotic use, and the encouragement of patient education and engagement^2^. Due to this lack of knowledge and awareness, patients may engage in behaviours that increase their risk of nosocomial infections.

Furthermore, patients' perceptions of nosocomial infections, as well as their attitudes toward infection prevention and control, can have an impact on the prevalence of these infections. A study in a teaching hospital in Nigeria, for example, discovered that patients' perceptions of the cleanliness of the hospital environment and the quality of care they received were significant predictors of their willingness to comply with infection prevention and control measures^14^. Understanding patients' knowledge and perceptions of nosocomial infections is therefore critical in developing effective strategies to reduce their occurrence.

Studies about knowledge and perception of nosocomial infections have mostly been done among health care professionals globally^15^ and in Nigeria^9,16^. There are limited studies on the assessment of knowledge and perception of nosocomial infections among patients in Nigeria. The study on the knowledge and perception of nosocomial infections among patients in a Nigerian tertiary hospital is critical for improving patient outcomes and lowering the country's burden of healthcare-associated infections.

Assessing patients' knowledge and perceptions of nosocomial infections in a tertiary hospital is vital for several reasons. It empowers patients through better education, ensuring they understand and actively participate in infection prevention measures. Informed patients are more likely to follow hygiene protocols, reducing the spread of infections within the hospital, benefiting both patients and healthcare staff. This study also contributes to improved healthcare quality and patient safety by identifying areas of concern warranting intervention.

Additionally, it holds financial implications, as the prevention of nosocomial infections leads to cost savings for healthcare facilities through shortened hospital stays and decreased readmissions. The results inform research, policy development, and initiatives to enhance quality, ultimately creating a safer and more patient-focused healthcare environment.

A tertiary hospital such as Federal Medical Centre is likely to have a higher burden of nosocomial infections than secondary or primary healthcare facilities due to the complex nature of medical care provided. Therefore, this study to assess the knowledge and perception of nosocomial infections was done among patients in Federal Medical centre, Ebute-Metta, Lagos.

## Methods

### Study area

Lagos state is situated in the South Western Nigeria within latitude 602′N to 604′N and longitude 2045′E to 4020′E The state is bounded from the North and East by Ogun State, in the West by the Republic of Benin and the South by the Atlantic Ocean. The total land mass of the state stretches over 3345 kms with an estimated population of about 15 million. About 40% of the total land area in the state is covered by water and wetlands^17^.

### Research design

A cross sectional research design was employed in this research.

### Study population

The study population were in-patients attending Federal medical Centre, Ebute-Metta, Lagos.

### Inclusion and exclusion criteria

All patients aged 18 years and above, who gave consent, were enrolled into the study while patients that were too ill and who did not give consent to participate were excluded.

### Sample size determination

The population was estimated from the average number of inpatients admitted into all wards per week = 10.

The total number of wards used in the study = 10 wards.

Total estimated population of patients admitted to all wards per week $=10\times 10$ = 100 patients.

The study duration was for 3 weeks thus, estimated population = 300 patients.

Raosoft® online calculator was used for the calculation of the sample size at 95% confidence interval and 5% level of significance = 95.

Addition of 10% attrition rate = $95+9.5$  = 104.5 patients. Approximately 105 patients.

### Sampling technique

There were 12 wards in the hospital and 10 wards were accessible to carry out the study. The two wards that were not accessible were Isolation and Accident and Emergency units because of the hospital policies. The hospital policies included inconvenience to the patients in those units stating that the patients may not be in the right state or frame of mind to participate in the study. Consecutive sampling method was used to select respondents for the study in each ward. This hospital was chosen because it is a tertiary hospital which is likely to have a higher burden of nosocomial infections than secondary or primary healthcare facilities due to the complex nature of medical care provided. The obvious limitation, however, is that the study was carried out in one hospital and the number of patients enrolled were relatively small which could potentially affect generalizability of the findings.

### Instrument for data collection

The study was conducted using a 21-item interview-based semi-structured questionnaire designed based on the objectives of this study. The questionnaire had 3 sections. The first section had 6 items and it documented the socio-demographics of the patients such as age, gender and marital status while the second section had 7 questions to assess the knowledge of the participants on nosocomial infections. The questions were made up of yes and no as well as multiple choice questions. A score of “1” was allocated to each correct answer while “0” was given to each wrong answer. The patients’ knowledge was categorised into Good (< 50% of total score), Fair (50–69% of total score) and Poor (≥ 70% of total score). Section C assessed the perception of the patients on nosocomial infections using eight 4-point Likert scale questions where strongly agree had the highest rank and strongly disagree had the lowest. The patients’ perception was ranked based on the perception scores. The perception scores were converted into Z-scores where the scores above the mean score was grouped as positive perception and scores below the mean score was grouped as negative perception.

### Validity and reliability of the instrument

The face and content validity was ascertained by experts who are lecturers in the Department of Public Health, Afe Babalola University, Ado-Ekiti. The corrections were implemented and adjusted based on their recommendation. A pre-test was carried out among 10 patients in Ipakodo Primary Health Care Centre, Lagos which was excluded from the main study analysis. Cronbach alpha test was used to evaluate the instrument reliability. A score of 0.742 was obtained from the findings, showing good reliability.

### Data collection and data analysis

Data was obtained using an interview-based administered semi structured questionnaire. Patient eligibility was assessed based on study inclusion criteria after which a written informed consent was obtained before enrolment into the study. Results were analysed and expressed in percentages, frequencies, tables and charts for categorical variables. Chi-square test and Fisher-Freeman-Halton Exact Test were used to evaluate the relationship between knowledge, perception and associated factors. Independent sample T-test and one way Analysis of Variance was used to assess statistical difference between categorical variables with 2 and 3 groups respectively. Level of significance was set at 5%. All analysis was carried out using the statistical package for the social sciences (SPSS) version 27.0.

### Ethics approval and consent to participate

Ethical clearance was obtained from Federal Medical Centre, Ebute-Metta, Lagos Health Research Ethics Committee with approval number HREC 23-13 before the research was conducted. Patient eligibility was assessed based on study inclusion criteria after which a written informed consent was obtained before enrolment into the study. The confidentiality of the information they give was maintained. All methods were performed in accordance with the relevant guidelines and regulations.

## Results

### Demographics of study participants and patients’ knowledge of nosocomial infections

A total of 102 patients gave consent to participate in this study of which 46.1% (47) were male, 27.5% (28) were aged between 38 and 47 years and 69.6% (71) were married. Sixty-seven (65.7%) were Christians while 48.1% (49) and 15.1% (16) were of Yoruba and Hausa ethnicity respectively. This has been summarized in Table 1. During the assessment of patients’ knowledge of nosocomial infections, 23.5% (24) stated that they know what is meant by nosocomial infections. About 57% (58) identified the elderly as a group at risk of nosocomial infections while 34.3% (35), 73.5% (75), and 15.7% (16) stated that pregnant women, smokers and visitors do not belong to groups of people at risk of nosocomial infections respectively.

**Table 1:** Demographics of study participants.

Variable	Frequency (n = 102)	Percent
Age		
18–27 years	23	22.5
28–37 years	26	25.5
38–47 years	28	27.5
> 48 years	25	24.5
Gender		
Male	47	46.1
Female	55	53.9
Marital status		
Single	31	30.4
Married	71	69.6
Educational status		
Primary education	8	7.8
Secondary	41	40.2
Tertiary	53	52.0
Ethnicity		
Yoruba	49	48.0
Igbo	37	36.3
Hausa	16	15.7
Religion		
Christianity	67	65.7
Islam	35	34.3

Majority [93.1% (95)] reported that poor hospital hygiene is a risk factor of nosocomial infection while 26.5% (27) identified invasive nonsurgical procedure as a risk factor. More than 70% (73) reported that healthcare workers who do not wear mask during procedures and patient care activities run a risk of coming down with nosocomial infections while 5.9% (6) did not know if healthcare workers who do not wear gloves when touching mucous membranes can increase risk of nosocomial infections. About 96% (98) stated that fully disinfecting the skin and equipment is a preventive measure against nosocomial infections while 49% (50) did not believe that wearing protective equipment like facemasks and gloves can prevent nosocomial infections. About 54% (55) believed that all nosocomial infections are preventable while all patients identified unsterilized equipment as a mode of spreading nosocomial infections. Overall, 15.7% (16) of the participants showed good knowledge while 12.7% (13) had poor knowledge of nosocomial infections. This has been summarized in Table 2.

**Table 2:** Patients’ knowledge of nosocomial infections.

S/N.	Statement	Responses (%)		
		Yes	No	I don’t know
1	Do know about what is meant by nosocomial infections?	24 (23.5)	49 (48.0)	29 (28.5)
2	Groups at risk of nosocomial infection			
	Elderly	58 (56.9)	15 (14.7)	27 (26.4)
	Children	38 (37.3)	44 (43.1)	20 (19.6)
	Pregnant women	45 (44.1)	35 (34.3)	22 (21.6)
	Smokers	17 (16.7)	75 (73.5)	10 (9.8)
	Visitors	76 (74.5)	16 (15.7)	10 (9.8)
	Patients in surgical wards	99 (97.0)	1 (1.0)	2 (2.0)
3	Risk factors of nosocomial infection			
	Poor Hospital hygiene	95 (93.1)	2 (2.0)	5 (4.9)
	Invasive nonsurgical procedure	27 (26.5)	29 (28.4)	46 (45.1)
	Length of hospital stay	71 (69.6)	10 (9.8)	21 (20.6)
4	Healthcare worker behaviour that can increase risk of nosocomial infection			
	The healthcare workers who do not wear gloves when touching mucous membranes (e.g. nose, mouth, etc.) or non-intact skin	93 (91.2)	3 (2.9)	6 (5.9)
	Healthcare workers who do not wear mask during procedures and patient-care activities	73 (71.6)	9 (8.8)	20 (19.6)
	Healthcare workers who do not wear gloves and mask during invasive nonsurgical procedures	67 (65.7)	3 (2.9)	32 (31.4)
5	Which of the following are preventive measures against nosocomial infections?			
	Fully disinfecting skin and equipment	98 (96.0)	1 (1.0)	3 (3.0)
	Washing hands occasionally	98 (96.0)	2 (2.0)	2 (2.0)
	Wearing protective equipment like face masks and gloves	94 (92.2)	5 (4.9)	3 (2.9)
	Regularly changing urinary catheters, and removing them as soon as possible	35 (34.3)	36 (35.3)	31 (30.4)
	Patients wearing goggles while in the surgical ward	9 (8.8)	50 (49.0)	43 (42.2)
	Prescribing antibiotics only when demanded by the patient	8 (7.8)	45 (44.2)	49 (48.0)
6	Do you believe all hospital acquired infections are preventable?	55 (53.9)	20 (19.6)	27 (26.5)
7	Do you understand how infections are spread in the hospital?	29 (28.4)	22 (21.6)	51 (50.0)
8	What are the potential modes of spread of hospital acquired infections?			
	Airborne	43 (42.2)	33 (32.3)	26 (25.5)
	Dirty beds	70 (68.6)	17 (16.7)	15 (14.7)
	Unsterilized equipment	102 (100)	–	–
	Improper wound dressing	101 (99.0)	1 (1.0)	–

Score	Frequency (%)	Remark		
< 50%	13 (12.7)	Poor knowledge		
50–69%	73 (71.6)	Fair knowledge		
≥ 70%	16 (15.7)	Good knowledge		
Mean knowledge score ± SD (range): 15 ± 2.599 (4–21) Total obtainable score: 25				

### Perception of patients towards nosocomial infections

Most patients 51% (52) in this study showed a negative perception of nosocomial infections. About 40% (38) strongly agreed that making alcohol rubs for all visitors mandatory would decrease the incidence of nosocomial infections while 23.5% (24) strongly disagreed that limiting visiting hours would minimize the risks of hospital acquired infections. About 6% (6) agreed that telling your healthcare provider if previously infected is necessary while all patients strongly agreed that if left untreated, these infections can become life threatening. This has been summarized in Table 3

**Table 3:** Patients’ perception of nosocomial infections.

S/N.	Statement	Responses (%)				
		SA	A	D	SD	Median
1	Making alcohol rubs for all visitors mandatory would decrease the incidence of hospital acquired infections	38 (37.3)	26 (25.5)	19 (18.6)	19 (18.6)	A
2	Limiting visiting hours would minimize the risks of hospital acquired infections	31 (30.4)	25 (24.5)	22 (21.6)	24 (23.5)	A
3	Restricting patients movement from ward to ward will decrease the risks of hospital acquired infections	50 (49.0)	18 (17.6)	14 (13.7)	20 (19.6)	A
4	Better patients compliance with infection control measures(hand hygiene) will decrease the risks of hospital acquired infections	80 (78.4)	18 (17.6)	–	4 (3.9)	SA
5	Taking all of your antibiotics as directed after discharge and completing the dose is important	74 (72.5)	17 (16.7)	5 (4.9)	6 (5.9)	SA
6	If infected, not sharing personal items like towels and razors is important	90 (88.2)	11 (10.8)	1 (1.0)	–	SA
7	If infected previously, telling your healthcare provider is necessary	96 (94.1)	6 (5.9)	–	–	SA
8	If left untreated, these infections can become life threatening	102 (100)	–	–	–	SA
	Mean perception score ± SD (range): 27.471 + 3.101 (21–32)					
	Positive perception	50 (49.0)				
	Negative perception	52 (51.0)				

*SA* Strongly Agree-4, *A* Agree-3, *D* Disagree-2, *SD* Strongly Disagree-1.

### Relationship between patient demographics, knowledge and perception of nosocomial infections

Differences in knowledge based on patient demographics, as well as the association between patient demographics and perception of patients on nosocomial infections were explored. There was no statistically significant difference in knowledge scores based on gender (p = 0.820) but there was a statistically significant difference based on age (p = 0.012). There was also no significant difference in knowledge scores based on religion (p = 1.000), educational status (p = 0.209) and ethnicity (p = 0.390) (Table 4). There was a statistically significant association between age and perception of patients towards nosocomial infections (p = 0.031). There was also no significant association between gender (p = 0.435), marital status (p = 0.393), religion (p = 0.098), and perception of the patients towards nosocomial infections (Table 5).

**Table 4:** Differences in knowledge scores on nosocomial infections based on patient demographics.

Variables	Frequency (%)	Mean score ± SD	p-value
Gender			
Male	47 (46.1)	15.06 ± 2.574	0.820^a^
Female	55 (53.9)	14.95 ± 2.642	
Marital status			
Single	31 (30.4)	14.90 ± 3.429	0.805^a^
Married	71 (69.6)	15.04 ± 2.168	
Age			
18–27 years	23 (22.5)	13.96 ± 3.007	0.012^b,^*
28–37 years	26 (25.5)	16.11 ± 2.389	
38–47 years	28 (27.5)	15.39 ± 2.424	
≥ 48 years	25 (24.5)	14.36 ± 2.139	
Religion			
Christianity	67 (65.7)	15.00 ± 2.564	1.000^a^
Islam	35 (34.3)	15.00 ± 2.701	
Educational status			
Primary	8 (7.8)	13.50 ± 1.852	0.209^b^
Secondary	41 (40.2)	14.98 ± 1.968	
Tertiary	53 (52.0)	15.25 ± 3.044	
Ethnicity			
Yoruba	49 (48.1)	14.89 ± 2.874	0.390^b^
Hausa	16 (15.7)	14.38 ± 2.655	
Igbo	37 (36.3)	15.41 ± 2.153	

*SD* Standard deviation; ^a^Independent Sample T-test; ^b^One Way Analysis of Variance; *Statistically significant value, p ≤ 0.05.

**Table 5:** Association between demographics and patients’ perception of nosocomial infections.

Variables	Perception		X^2^	p-value
	Negative	Positive		
Gender				
Male	26	21	0.657	0.435^a^
Female	26	29		
Marital status				
Single	18	13	0.894	0.393^a^
Married	34	37		
Age				
18–27 years	13	10	8.843	0.031^a,^*
28–37 years	10	16		
38–47 years	20	8		
≥ 48 years	9	16		
Religion				
Christianity	30	37	3.008	0.098^a^
Islam	22	13		
Educational status				
Primary	4	4	0.804	0.690^b^
Secondary	23	18		
Tertiary	25	28		
Ethnicity				
Yoruba	24	25	4.659	0.098^a^
Hausa	16	21		
Igbo	12	4		

^a^Pearson Chi-square test; ^b^Fisher–Freeman–Halton Exact Test; *Statistically significant value at p ≤ 0.05.

## Discussion

### Knowledge of patients on nosocomial infections

Nosocomial infections are a major public health concern worldwide, causing significant morbidity and mortality among hospitalized patients in developing countries including Nigeria^18^. This rise in prevalence is also associated with higher healthcare costs and longer hospital stays. The prevalence of nosocomial infections has been reported to be as a result of poor knowledge and perception of patients^13^.

The patients identified various modes of transmission of nosocomial infections such as airborne, dirty beds, unsterilized equipment, and improper wound dressing. This is slightly different from findings by Khan and colleagues who stated that nosocomial infections can be spread from person to person, via shared objects and surfaces, the environment, contaminated water and food, diseased people, and contaminated healthcare workers' skin^6^. This simply illustrates the ubiquity of these infections and ease of spread.

In this study, majority of the patients showed fair to poor knowledge of nosocomial infections. This is in contrast with findings from the study carried out by Ocran and Tagoe, where the knowledge shown by the patients were deemed adequate^19^. This could be due to over 48% of the participants having completed primary and secondary education. There is a need to improve knowledge of patients regarding nosocomial infections.

This study also recorded higher average knowledge among the male patients than in the female patients. The higher knowledge among the male patients in this study could be due to the higher tendency of males to come down with nosocomial infections than females^20,21^. This is in contrast to findings by Rahiman et al. where females had a higher knowledge score than males, although the study was carried out among nursing students^22^.

Findings in this study also revealed that patients with higher education also displayed better knowledge of nosocomial infections than those with a lower level of education. This shows the importance of proper education on knowledge of nosocomial infections. Thus, hospital associated infections may decline as a result of increased knowledge of hospital associated infections and adherence to preventative measures. A higher education level may positively influence both patient health education and their ability to pay attention to and understand educational materials^23^.

There was a higher level of knowledge shown among the participants with higher age in this study. This is similar to findings from a studies carried out in Ethiopia^24^ and Ghana^25^ where older age was associated with higher knowledge among the study participants. Although the population in the two studies were healthcare workers who often receive specialised training on nosocomial infections. The possible reason for a high level of knowledge shown by older patients in this study could be the risk of nosocomial infections. Several studies have reported a significant relationship between increasing age and risk of developing nosocomial infections^26,27^. Lower mean knowledge scores were also reported among patients aged 18–28 years. Thus, interventions in the form of education and awareness campaigns should be carried out targeted towards younger patients.

Patients who were married had higher knowledge scores than those single which is in tandem with findings from a study in Ethiopia where married participants had a higher likelihood to have better knowledge of nosocomial infections than single^24^. This could be due to the fact that married people tend to have better health seeking behaviour. Marital status can have a positive influence on health-seeking behaviour^28^. Although, the impact of marriage on health-seeking behavior is highly individual and can be influenced by various factors.

### Patients’ perception of nosocomial infections

Additionally, majority of the patients showed a negative perception towards nosocomial infections as observed in this study. This is in contrast with the study in UK by Smyth et al.^29^. In India, Jeyasheelan and colleagues reported that the participants showed a high perception towards nosocomial infections^30^. There was a significant association between age and perception towards nosocomial infections in this study and patients whose age was above 48 years had a more positive perception of nosocomial infections than those below 48 years. This could be due a higher prevalence of nosocomial infections among patients in the older age group^31^. Intervention on nosocomial infections should be targeted more towards patients of the older population to help improve their perception.

Furthermore, the study also revealed that making alcohol rubs, restricting patients’ movement from ward to ward and use of control measures such as hand hygiene can bring about a decrease in nosocomial infections. This is similar to findings by Smyth et al. where the use of alcohol-based hand rub for visitors (67%), stopping the movement of patients from ward to ward (45%), and restricting the number of visitors (18%) were suggested measures by the patients^29^.

### Practical implications of study findings

In this study, the participants showed fair knowledge about nosocomial infections coupled with negative perceptions which could have far-reaching implications for healthcare policy and patient education.

Healthcare policies can be customized to specifically address these concerns, emphasizing the need for improved infection prevention measures and more stringent regulatory standards. These policies may also establish patient rights related to infection control and healthcare providers' responsibilities to educate patients about infection risks.

Patient education programs need to be tailored to dispel misconceptions and alleviate negative perceptions. Clear and accurate information should be provided, empowering patients to actively engage in their care and infection prevention. Furthermore, improved communication strategies, feedback mechanisms, and transparency initiatives are essential to rebuilding trust and addressing patient concerns.

### Limitations

This study was carried out in one centre/hospital and the findings might not be generalizable to other health institutions in Nigeria. However, the findings in this study provide information on the state of knowledge and perception of patients towards nosocomial infections and could serve as background or platform for future studies. Also, the patients’ knowledge and perception was not analysed based on patient wards which could have provided more information on the state of knowledge of the patients on nosocomial infections.

## Conclusion

This study showed that most patients had fair knowledge as well as negative perception towards nosocomial infections. Also, certain factors were assessed for their potential influence on patients’ knowledge and perception towards nosocomial infections but age, had influence on patients’ perception and knowledge of nosocomial infection. There is a need for educational intervention and awareness targeted towards certain age groups to improve knowledge and perception of patients towards nosocomial infections.

## Data Availability

The datasets used and/or analysed during the current study are available from the corresponding author on reasonable request.

## Abbreviation

WHO: World Health Organization
